# Association of Veterinary Faculties of North America

**Published:** 1896-10

**Authors:** H. D. Gill

**Affiliations:** Secretary


### ASSOCIATION OF VETERINARY FACULTIES OF NORTH AMERICA.

THE fourth meeting of the Association was held at the University of Buffalo, High Street, Buffalo, September 4, 1896, the following members being present: Drs. Lyman and Osgood, of Harvard University; Dr. McEachran, of McGill University; Dr. Smith, of Ontario Veterinary College; Drs. R. R. Bell and James L. Robertson, of the American Veterinary College; Dr. Leonard Pearson, of the University of Pennsylvania; Dr. D. E. Salmon, of the National Veterinary College (Columbian University); Dr. Hughes, of the Chicago Veterinary College; Dr. Jas. M. Wright, of McKillip Veterinary College; Dr. Stalker, of the Iowa Agricultural College; Dr. Stewart, of the Kansas City Veterinary College: Drs. Law and Williams, of Cornell University; Dr. Gill, of the New York College of Veterinary Surgeons; and Dr. W. H. Hoskins, President of the United States Veterinary Medical Association. President Lyman was in the chair and Dr. Gill acted as Secretary.

After roll-call, the President, in a short address, recited the history of and progress made by the Association. The minutes of the previous meeting were read, and, on motion of Dr. Salmon, seconded by Dr. Osgood, were accepted. The Secretary-Treasurer's report being read, on motion of Dr. Osgood, seconded by Dr. McEachran, was approved and accepted.

The next order of business was the reading of the papers. First subject "Uniform Course of Instruction," Dr. J. W. Adams, of the University of Pennsylvania; but the speaker not being present, was laid over. The second subject, that of "Mutual Recognition of Students by Colleges Belonging to the Association of Veterinary Faculties," was then in order, and a paper on that topic was read by Dr. H. D. Gill, and discussed by Drs. Law, Pearson, Stalker, Salmon, McEachran, Hughes, Lyman, and Wright. A motion by Dr. Pearson, seconded by Dr. Osgood, that a committee of three be appointed to consider the question and to report in thirty minutes, was amended by Dr. Stalker, seconded by Dr. Salmon, that the committee report at the next annual meeting, instead of in thirty minutes. This motion was again amended by Dr. Salmon, seconded by Dr. Stalker, that a minimum standard of preliminary examinations be recommended. Carried as amended. The President appointed Drs. Law, Salmon, and Pearson. The third subject, that of "Competitive Examinations for Positions in the Veterinary Faculties," was, on motion of Dr. Pearson, seconded by Dr. Salmon, laid on the table. The fourth subject, that of "College Fees," was, on motion of Dr. Salmon, seconded by Dr. Law, laid on the table.

The next in order was the admission of members—Drs. Law, Williams, Smith, McEachran, Hughes, and Wright signing the Constitution.

The next order of business was the nomination and election of officers. For President Dr. Pearson was nominated by Dr. Osgood, seconded by Dr.

Bell, and, on motion, the Secretary cast the ballot, Dr. Pearson being declared elected. For Secretary-Treasurer Dr. Gill was nominated by Dr. Osgood, seconded by Dr. Salmon, and was elected, the President casting the ballot. Nominations for Executive Committee: Dr. Law, nominated by Dr. Stalker, seconded by Dr. Osgood; Dr. Stalker, nominated by Dr. Bell, seconded by Dr. Osgood; Dr. McEachran, nominated by Dr. Osgood, seconded by Dr. Bell; Dr. Salmon, nominated by Dr. Osgood, seconded by Dr. Gill; Dr. Robertson, nominated by Dr. Bell, seconded by Dr. Gill. It was regularly moved and seconded that the Secretary cast a ballot for each of the Executive Committee. Carried. The Secretary casting the ballots, the President then declared the above-named gentlemen duly elected members of the Executive Committee.

Under the head of new business the question of a National Board of Veterinary Examiners was brought up and discussed by Drs. Salmon, Pearson, Hoskins, Law, and Stalker, and a motion of Dr. Pearson, seconded by Dr. Gill, that Dr. Hoskins be requested by this Association to use his efforts on behalf of this Association to procure uniformity in future laws regulating the practice of veterinary medicine in the various States of the United States and secure the introduction of a clause in such laws that will enable the Boards to be established to co-operate with the National Association and secure a uniform examination, and that Dr. Hoskins be requested to report upon this matter at the next annual meeting. Carried.

On motion of Dr. Osgood, seconded by Dr. Gill, Dr. Hoskins was elected an honorary member.

In a very neat little speech President Lyman spoke of the confusion of the meeting, and suggested that hereafter the Association should have a day and time of its own, so as not to have its business interfered with. On motion of Dr. Osgood, seconded by Dr. Pearson, the meeting adjourned.

H. D. Gill,
Secretary.

